# Could the Novel Oral Anticoagulants Be Used for Coronary Artery Aneurysm?

**DOI:** 10.1155/2020/5073814

**Published:** 2020-04-25

**Authors:** Quanxiang Yan, Libing Ning, Yu Jian, Wencong Yang, Qinghua Yuan, Zhimin Du

**Affiliations:** ^1^Cardiovasology, The Seventh Affiliated Hospital of Sun Yat-sen University, Shenzhen, Guangdong, China; ^2^Department of Cardiology, The First Affiliated Hospital of Sun Yat-sen University, Guangzhou, Guangdong, China

## Abstract

Coronary artery aneurysms (CAAs) are uncommon in coronary angiography, and left main coronary artery aneurysms are rare. There is no consensus for the treatment of CAAs. A young patient with left coronary artery aneurysm diagnosed by coronary angiography and with recurrent acute myocardial infarction was treated with rivaroxaban and aspirin. The patient had no angina for 6 months. Novel oral anticoagulants combined with antiplatelet agents may be appropriate for the treatment of CAAs.

## 1. Introduction

Coronary artery aneurysms (CAAs) are uncommon in coronary angiography (CAG), and left main coronary artery (LMCA) aneurysms are rare [1]. There is no consensus for the treatment of CAAs [2]. We present a case of CAAs involving the LMCA and the left anterior descending (LAD) artery with recurrent myocardial infarction. The patient treated with rivaroxaban and aspirin had no angina for 6 months.

## 2. Case Presentation

A 33-year-old man was admitted to our hospital due to persistent chest pain for 14 hours, without dyspnea and syncope. He occasionally smokes and denied of having hypertension, diabetes, and dyslipidemia. After admission, the patient still complained of having chest pain. Physical examination revealed the blood pressure of 106/79 mmHg and the heart rate of 70 bpm (regular). No cardiac murmur and rales were heard.

**Figure 1:**
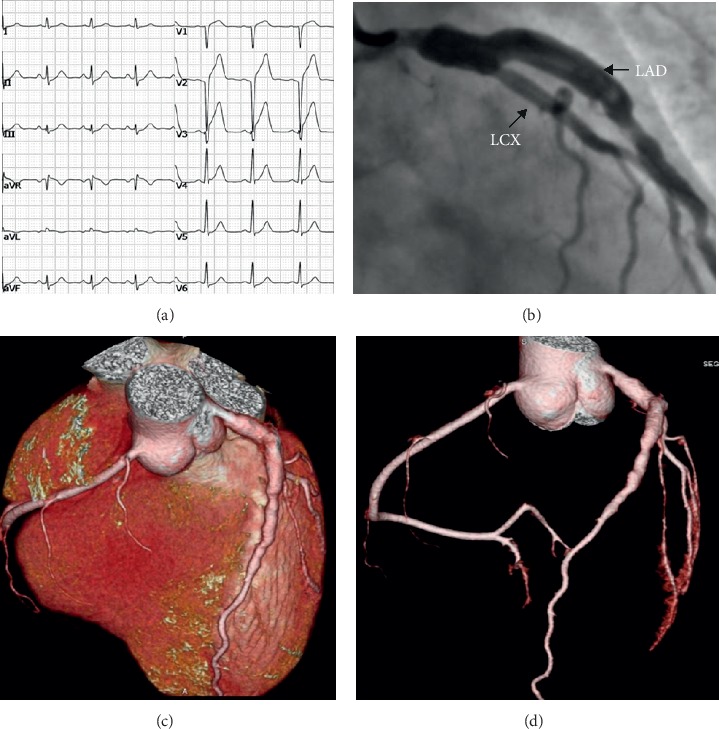
(a) ECG on admission: normal sinus rhythm with ST segment elevation on leads V1–3. (b) CAG: an aneurysmal dilatation from the LMCA to the proximal segment of LAD with a transverse diameter of 8.5 mm. (c, d) Coronary CTA with three-dimensional volume rendering: multiple aneurysms from the LMCA to the proximal segment of the LAD artery.

Admission laboratory tests revealed cTn-I of 5.5284 ng/ml (first time, normal value <0.03 ng/ml), CK-MB of 61.9 ng/ml, and cTn-I of 26.35 ng/ml (peak level). His electrocardiogram (ECG) revealed normal sinus rhythm with ST segment elevation on leads V1–3 (Figure 1(a)). The primary impression was acute ST segment elevation myocardial infarction (Killip class I). And then, emergency coronary angiography (Figure 1(b)) revealed the right coronary artery (RCA) and the left circumflex (LCX) had no significant stenosis with a forward flow of TIMI level 3, and there was an aneurysmal dilatation from the LMCA to the proximal segment of LAD with a transverse diameter of 8.5 mm, and the middle and distal segments of LAD had no obvious stenosis with a forward flow of TIMI level 3. To further assess CAAs, we performed contrast-enhanced coronary artery computer tomography angiography (CTA) with three-dimensional reconstruction of coronary arteries (Figures 1(c) and 1(d)).

The final diagnosis was ST segment elevation acute myocardial infarction (Killip class I), coronary artery aneurysms. During his hospitalization, the patient was treated with low-molecular-weight heparin and dual antiplatelet agents (aspirin and clopidogrel) for a week, and then, rheumatoid immune diseases and vasculitis were excluded. The patient was discharged from the hospital with aspirin and clopidogrel.

One month later, the patient was readmitted to our hospital with recurrent acute myocardial infarction. But, the CAG was declined by the patient. Based on his medical history, ECG, and laboratory tests, we considered that the myocardial infarction was due to coronary aneurysm again. Then, he was treated with rivaroxaban (20 mg once daily) and aspirin. Rivaroxaban is one of the novel oral anticoagulants (NOACs). The patient did not complain of chest pain and was followed up for 6 months after discharge.

## 3. Discussion

Acute myocardial infarction is widespread in the elderly, and the main etiology was coronary atherosclerosis. CAAs refer to the limitation or diffuse dilatation of the coronary artery, and generally, diameter exceeds 1.5 times of the adjacent normal coronary artery [3]. The morbidity of CAAs is about 0.3%–4.9%, and CAAs mostly occur in the RCA. LMCA aneurysms are extremely rare and are accounted for approximately 0.1% of the coronary artery aneurysms [4, 5]. Potential complications of CAAs include rupture, thrombosis, embolization, dissection, mechanical obstruction, and erosion into surrounding structures [6]. Causes of coronary aneurysms include arteriosclerosis, autoimmune diseases, congenital defects, infections, and complications of percutaneous coronary intervention.

No consensus has been established regarding optimal management of CAAs. Treatment strategies include surgical approach, percutaneous covering stenting, and medical management [2]. Surgical intervention may be considered when the aneurysm dilates more than 2.5 cm, when there is an intramural thrombus, or when there are complications such as fistula formation, compression of the cardiac chambers, and rapidly increasing size of the aneurysm [2, 7, 8]. CAAs with a diameter less than 10 mm, a straight lesion vessel, and located in the proximal vessel may benefit more from stenting [9]. For medical management, some scholars have concluded that warfarin combining with aspirin can reduce the incidence of coronary occlusion, myocardial infarction, and death in children with giant CAAs caused by Kawasaki disease [10].

The management of CAAs is often based on the onset of the disease, the patient's motivation, the doctor's advice, and relevant guidelines or literature. In this case, the CAA was dilated from the LMCA to the LAD, diameter of 8.5 mm, a fusiform shape, and length of over 30 mm. So, we considered medical management for the patient. Thrombosis is one of the main causes of myocardial infarction in CAAs. Ispas et al. [11] used aspirin combined with warfarin for the patients with left main aneurysm stents at long durations. A recent study also suggested a possible advantage of anticoagulation in patients with CAAs and acute coronary syndrome [12]. In this case, the patient refused warfarin. Worried about the thrombosis, we tried rivaroxaban combining with aspirin. The patient was discharged from the hospital and was followed up for 6 months. There was no recurrence of chest pain or myocardial infarction. The efficacy and safety of NOACs are superior to warfarin in patients with nonvalvular atrial fibrillation [13, 14]. By searching the literature, there are few cases of the application of NOACs. The cases of treating CAAs with NOACs are shown in Table 1. After analyzing these literature studies, we found that the antithrombotic strategies and dosages were varied widely. And for this patient, normal doses of rivaroxaban (20 mg once daily) and aspirin are also suitable for the treatment of CAAs. Further clinical trials are needed to provide stronger evidence upon this matter.

## 4. Conclusion

By reviewing the relevant literature and analyzing the diagnosis and treatment of CAAs, we suggested that NOACs combined with antiplatelet agents may be suitable for the treatment of CAAs.

## Figures and Tables

**Table 1:** The cases of treating CAAs with NOACs.

Author-first	Publication year	Location of CAAs	Type of anticoagulants	Antiplatelet agents	Follow-up time (month)
Grigorios et al. [15]	2017	RCA	Rivaroxaban	Aspirin^a^, clopidogrel	>3
Choi et al. [16]	2018	RCA, LAD, LCX	Rivaroxaban	Clopidogrel	11
Yuksel et al. [17]	2015	Coronary thrombosis	Rivaroxaban	—	2
Waqas et al. [18]	2019	RCA, LAD, LCX	Warfarin followed by rivaroxaban	Ticagrelor, aspirin	—
Tomioka et al. [19]	2016	RCA	Warfarin followed by apixaban	Aspirin	12

^a^Stopped after one month.
